# TMB‐High, MSI‐High Castration‐Resistant Prostate Cancer Treated With Pembrolizumab

**DOI:** 10.1002/iju5.70062

**Published:** 2025-06-20

**Authors:** Satoshi Muraoka, Hisanobu Tosuji, Yuya Iwahashi, Hiroki Kawabata, Ryusuke Deguchi, Takahito Wakamiya, Shimpei Yamashita, Yasuo Kohjimoto, Isao Hara

**Affiliations:** ^1^ Department of Urology Wakayama Medical University Wakayama City Japan

**Keywords:** microsatellite instability‐high, pembrolizumab, prostate cancer, tumor mutation burden‐high

## Abstract

**Introduction:**

The use of pembrolizumab in patients with microsatellite instability‐high (MSI‐high) and tumor mutation burden‐high (TMB‐high) prostate cancer in Japan is not widely reported. Here, we report the case of a patient with MSI‐high and TMB‐high prostate cancer who responded well to pembrolizumab after multiple systemic treatments.

**Case Presentation:**

A 68‐year‐old Japanese man was diagnosed with cT4N1M1a prostate cancer. He was treated with several androgen receptor signaling inhibitors and chemotherapy. After intense systemic treatment, disease progression was confirmed, and genomic testing detected MSI‐high and TMB‐high. However, treatment with pembrolizumab resulted in marked prostate‐specific antigen reduction and significant shrinkage of metastases.

**Conclusion:**

Genomic tests should be considered for high‐grade tumors. MSI‐high and TMB‐high prostate cancer responded well to pembrolizumab in this case, but patients should be carefully monitored for the development of side effects after administration of pembrolizumab.

**Summary**

- A 68‐year‐old man with MSI‐high and TMB‐high metastatic castration‐resistant prostate cancer received pembrolizumab. The volumes of lymph node metastasis were remarkably reduced and continued to shrink after its discontinuation. This case suggests that genomic tests should be considered for metastatic castration‐resistant prostate cancer.

**Abbreviations and Acronyms**
ARSI: androgen receptor signaling inhibitor
mCRPC: metastatic castration‐resistant prostate cancer
MSI‐high: microsatellite instability‐high
TMB‐high: tumor mutation burden‐high

## 1 Introduction

Pembrolizumab was approved in Japan for microsatellite instability‐high (MSI‐high) and tumor mutation burden‐high (TMB‐high) cancers in 2018 and 2022, respectively. Meanwhile, the use of pembrolizumab in the treatment of patients with prostate cancer in Japan is not widely reported. Here, we report the case of a patient with MSI‐high and TMB‐high prostate cancer that received effective pembrolizumab after multiple unsuccessful systemic treatments.

## 2 Case Report

**FIGURE 1:**
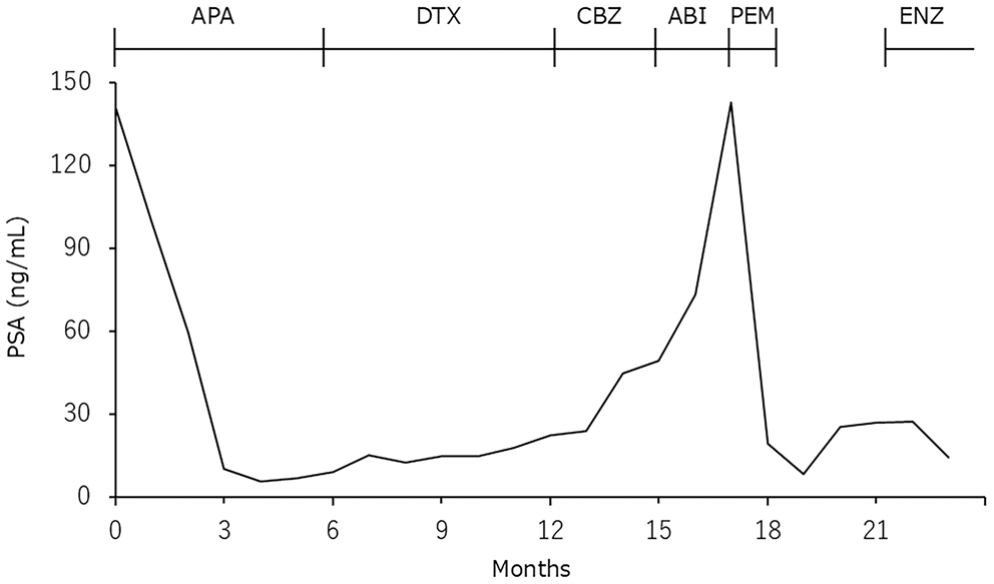
Prostate‐specific antigen values along the patient's clinical course. ABI, abiraterone; APA, apalutamide; CBZ, cabazitaxel; DTX, Docetaxel; ENZ, Enzalutamide; PEM, pembrolizumab; PSA, prostate‐specific antigen.

**FIGURE 2:**
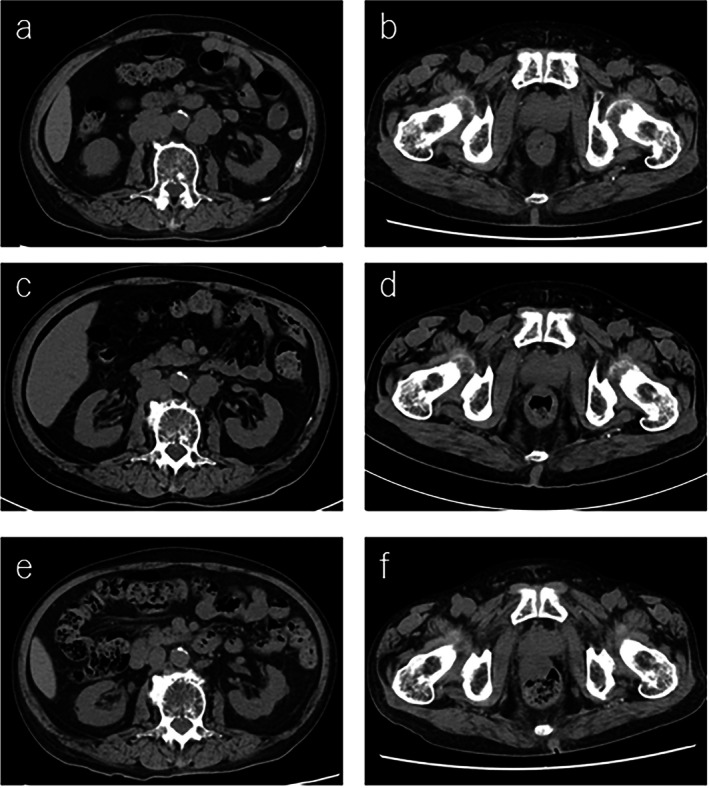
Images of abdominal contrast–enhanced computed tomography before (a, b), 1 month after (c, d), and 3 months after (e, f) the start of pembrolizumab treatment. (a, c, e) Images show a remarkable reduction in the size of the para‐aortic lymph node metastasis despite being taken 2 months after discontinuing pembrolizumab. (b, d, f) Images show tumor growth in the prostate region 2 months after pembrolizumab discontinuation.

A 68‐year‐old Japanese man was diagnosed with prostate cancer (initial prostate‐specific antigen [PSA] 141 ng/mL, adenocarcinoma, Gleason score 5 + 5, cT4N1M1a). He had no personal or family history of malignancy. He was treated with apalutamide and androgen deprivation therapy (ADT) for metastatic castration‐sensitive prostate cancer (mCSPC). PSA decreased from 141 to 5.5 ng/mL after 4 months but began to rise again, so docetaxel was started 7 months after diagnosis, when the PSA was 15.2 ng/mL. Despite sequential treatment with docetaxel (eight cycles), cabazitaxel (three cycles), and abiraterone acetate (1 month), the PSA increased to 143 ng/mL. Computed tomography showed increased lymph node metastasis in the para‐aortic region. Foundation One CDx of prostate biopsy specimens at the time of initial diagnosis showed the tumor was MSI‐high and also TMB‐high (53Muts/Mb), so treatment was switched to pembrolizumab. After one course of treatment, there was a marked decrease in PSA from 143 to 19.3 ng/mL, and there was shrinkage of para‐aortic lymph node metastasis. Pembrolizumab was discontinued due to drug‐induced liver injury (CTCAE grade 3). However, the following month, PSA was further reduced from 19.3 to 8.3 ng/mL. Two months after discontinuation of pembrolizumab, the PSA increased from 8.3 to 26.8 ng/mL, but the para‐aortic lymph node shrinkage was maintained while the primary tumor enlarged (Figures 1, 2). External radiation to the primary tumor and treatment with enzalutamide were therefore initiated, and PSA decreased to 14.1 ng/mL.

## 3 Discussion

In recent years, novel androgen receptor signaling inhibitor (ARSI) or docetaxel in combination with ADT has prolonged survival among patients with mCSPC. However, the median survival time after progression to mCRPC in Japanese patients is approximately 3 years [1], indicating that the disease has a poor prognosis.

In mCRPC patients after multiple treatments, responses are limited. In the phase III CARD trial, PSA50% response rates to another ARSI or cabazitaxel were 8.3% and 26.7%, respectively [2]. In contrast, pembrolizumab has shown a PSA50% response rate of around 50% in MSI‐high mCRPC [3, 4]. To date, nine cases of TMB‐high or MSI‐high prostate cancer have been reported in Japan (Table 1) [5, 6, 7, 8, 9, 10, 11], with most showing a significant PSA response and some experiencing clinical benefit beyond 18 months. These findings suggest that pembrolizumab may be highly effective even in heavily pretreated patients, highlighting the need for further study. In the current case, a marked PSA decrease and tumor shrinkage were seen after pembrolizumab administration following multiple treatments.

**TABLE 1:** Cases of pembrolizumab treatment for MSI‐high or TMB‐high prostate cancer in Japan.

Study (Year)	Age	Gleason score	Disease state (stage)	MSI	TMB	Past therapy	PSA at starting Pem (ng/mL)	PSA decline	Cycle number	Best response	Observation period (month)	Outcome
Fujiwara et al. (2020)	58	4 + 4	Metachronus CRPC	High	NE	RP, RT, CAB, ABI, ENZ, DTX, CBZ	16.6	63%	5 (undergoing)	PR	> 3	Alive
Kageyama et al. (2022)	70	4 + 4	Synchronus CRPC (stageD2)	High	NE	CAB, ENZ, ABI, DTX	about 250	> 90%	4	PR	> 9	Alive
Yoshida et al. (2022)	66	4 + 4	Synchronus CRPC (cT3aN0M1c)	High	NE	CAB, EP, CBDCA+CPT11	—	—	estimated 15 (undergoing)	CR	> 14	Alive
Shimizu K et al. (2022)	67	5 + 4	Synchronus CRPC (cT4N1M0)	High	High	Cab, ABI, DTX, CBZ, RT, TUR‐P	35.67	> 90%	estimated 25 (undergoing)	PR	> 18	Alive
Takasawa T, et al. (2024)	87	4 + 5	Metachronus CRPC	High	NE	RP, RT, CAB, ABI, ENZ, DTX, CBZ	408.78	83%	13	PR	11	Dead
Hirano T et al. (2024)	71	5 + 5	Synchronus CRPC (cT4N0M1b)	High	NE	ABI, RT, DTX, Ola	15.69	> 90%	6	PR	> 9	Alive
Yaegashi et al. (2024)	73	—	CRPC	Stable	High	—	—	—	—	—	1.5	—
	67	—	CRPC	High	High	—	—	—	—	—	1.5	—
	77	—	CRPC	Stable	High	—	—	—	—	—	—	—
Our case (2025)	69	5 + 5	Metachronus CRPC	High	High	APA, DTX, CBZ, ABI	143	> 90%	1	PR	4	Alive

Abbreviations: CAB, combined androgen blockade; CBDCA + CPT11, Carboplatin and Irinotecan; EP, Etoposide + Cisplatin; RP, radical prostatectomy; RT, radiation therapy; TUR‐P, transurethral resection of the prostate.

The frequency of MSI‐high and TMB‐high cases in prostate cancer is reported to be about 3%, respectively [1, 4]. Characteristics of MSI‐high prostate cancer include the high frequency of GS9‐10, neuroendocrine, and intraductal carcinoma [12]. Although the frequency of MSI‐high prostate cancer is not high, genomic testing is thought to be especially useful for the detection of high‐grade tumors, such as in the present case. Importantly, MSI‐high tumors warrant evaluation for Lynch syndrome, a hereditary cancer syndrome involving mismatch repair gene abnormalities. In one report, 16.3% of MSI‐high tumors were associated with Lynch syndrome [13]. In this case, loss of *MSH2* and *MSH6* frameshift mutation (G1139fs*6) were detected, and no direct copy number analysis or loss of heterozygosity assessment was performed. Genetic counseling was recommended, but it was not pursued.

A distinctive feature of this case is the concurrent elevation of TMB and MSI, likely contributing to the observed therapeutic response. Previous report has also shown overlap between these biomarkers: MSI‐high was detected in 2.2% of 1033 prostate cancers (all TMB‐high) [4]. In this case, FoundationOne testing identified *MSH2* and *MSH6* alterations, suggesting MSI‐high contributed to the elevated TMB.

In prostate cancer, TMB has been associated with older age, advanced T and N stage, and shorter overall survival [14]. Moreover, prostate cancer exhibits substantial genomic heterogeneity, with genetic variations observed across metastatic sites and within primary tumors [15, 16]. mCRPC displays more frequent genomic alterations and higher TMB than localized disease, which reflects increased genomic instability. TMB is reportedly greater in metastatic lesions and has been correlated with improved responses to pembrolizumab, likely due to an increased neoantigen load [17, 18]. These factors may collectively explain the site‐specific differences in treatment response observed in the present case.

In our patient, prostate radiotherapy demonstrated a therapeutic effect despite multiple lines of systemic therapy. Local radiotherapy to the primary tumor is established for low‐volume mCSPC and is recommended by guidelines (Japanese Prostate Cancer Clinical Practice Guideline 2023, NCCN guideline 2024, EAU guideline 2024). However, there is not currently robust evidence for its use in high‐volume mCSPC or mCRPC, and it is not currently recommended. Nevertheless, retrospective studies suggest potential benefits: one reported improved overall survival with prostate radiotherapy in mCRPC [19], another described durable disease control [20]. These findings suggest that local radiotherapy to the primary tumor may be a therapeutic option for selected mCRPC cases, warranting further large‐scale, prospective studies to refine patient selection.

There is currently no consensus on the optimal timing for administering immune checkpoint inhibitors (ICIs). Future studies may support earlier genomic testing and personalized treatment strategies. In Japan, however, comprehensive genomic profiling is typically performed only once after advanced CRPC has developed and is covered by insurance in that setting. Thus, performing this profiling after CRPC progression remains appropriate. Ongoing trials, such as the SBRT‐AMICO and NCT03951831, are evaluating the efficacy of anti‐PD‐1‐based combinations in mCSPC. While the optimal timing of ICI use in prostate cancer is still unclear, these studies may support earlier implementation and expanded indications.

Unfortunately, only one course of pembrolizumab was administered due to adverse events, but the lymph node metastases continued to shrink afterward, suggesting a lasting effect. However, the short observation period highlights the need for additional cases with longer follow‐up.

## 4 Conclusion

Genomic tests should be considered for high‐grade tumors. MSI‐high and TMB‐high prostate cancer responded well to pembrolizumab in this case, but patients should be carefully monitored for the development of side effects after administration of pembrolizumab.

## Disclosure

Approval of the Research Protocol by an Institutional Reviewer Board: N/A.

Registry and the Registration No. of the Study/Trial: N/A.

## Consent

Written informed consent to publication of this case report, including clinical details and clinical images, was obtained from the patient. A copy of the written consent form is available for review upon reasonable request.

## Conflicts of Interest

The authors declare no conflicts of interest.
